# Ambulatory Assessment of Instantaneous Velocity during Walking Using Inertial Sensor Measurements

**DOI:** 10.3390/s16122206

**Published:** 2016-12-21

**Authors:** Angelo Maria Sabatini, Andrea Mannini

**Affiliations:** The BioRobotics Institute, Scuola Superiore Sant’Anna, 56127 Pisa, Italy; andrea.mannini@santannapisa.it

**Keywords:** body center of mass, walking speed, inertial measurement unit, Fourier harmonic analysis, Bayesian methods, regression

## Abstract

A novel approach for estimating the instantaneous velocity of the pelvis during walking was developed based on Inertial Measurement Units (IMUs). The instantaneous velocity was modeled by the sum of a cyclical component, decomposed in the Medio-Lateral (ML), VerTical (VT) and Antero-Posterior (AP) directions, and the Average Progression Velocity (APV) over each gait cycle. The proposed method required the availability of two IMUs, attached to the pelvis and one shank. Gait cycles were identified from the shank angular velocity; for each cycle, the Fourier series coefficients of the pelvis and shank acceleration signals were computed. The cyclical component was estimated by Fourier-based time-integration of the pelvis acceleration. A Bayesian Linear Regression (BLR) with Automatic Relevance Determination (ARD) predicted the APV from the stride time, the stance duration, and the Fourier series coefficients of the shank acceleration. Healthy subjects performed tasks of Treadmill Walking (TW) and Overground Walking (OW), and an optical motion capture system (OMCS) was used as reference for algorithm performance assessment. The widths of the limits of agreements (±1.96 standard deviation) were computed between the proposed method and the reference OMCS, yielding, for the cyclical component in the different directions: ML: ±0.07 m/s (±0.10 m/s); VT: ±0.03 m/s (±0.05 m/s); AP: ±0.06 m/s (±0.10 m/s), in TW (OW) conditions. The ARD-BLR achieved an APV root mean square error of 0.06 m/s (0.07 m/s) in the same conditions.

## 1. Introduction

An important goal of locomotion is the displacement of the Body Center of Mass (BCOM), whose location in humans is somewhere within the pelvis. During walking, the pelvis moves in the three dimensional (3D) space, showing, relative to the mean forward velocity, speed fluctuations in the direction of progression [1].

One approach to investigate the behavior of the BCOM during gait consists of attaching a marker to the sacrum bone and tracking its motion using an Optical Motion Capture System (OMCS)—the sacral marker method [2]. In spite of being less accurate, this method is appealing for the ease of implementation, as compared with other approaches, such as the segmental analysis and the force plate methods [3]. However, a feature common to all these methods is that their application is restricted to gait labs [4]. Additionally, unless a treadmill is used to reproduce walkway conditions, the capture of a large sample of consecutive gait cycles is precluded [4]. Unobtrusive approaches to estimating the BCOM motion are thus needed, e.g., in the ambulatory assessment of external work during gait [5,6].

In this paper, we report and evaluate a novel inertial sensor-based algorithm that estimates the instantaneous velocity of an Inertial Measurement Unit (IMU) attached to the pelvis (pelvis IMU) during gait. For cyclical motions such as bipedal locomotion, the instantaneous velocity can be modeled by the sum of the 3D cyclical component of velocity and the velocity of forward motion averaged over each cycle, henceforth called the Average Progression Velocity (APV) [7,8]. It is commonplace to estimate the 3D cyclical component of velocity by numerical single time-integration of the linear acceleration, followed by high-pass filtering [8,9]. Recently, a Fourier-based method was proposed for analytical double time-integration of the linear acceleration of points on the human body moving cyclically [10]; this method, for which implementation one pelvis IMU and an additional IMU attached to one shank (shank IMU) were suggested, performed better than existing methods as for the closeness of agreement between the 3D displacements estimated by the pelvis IMU and the OMCS reference. In this paper, the Fourier-based approach to analytical integration was applied and validated for estimating the 3D cyclical component of pelvis velocity.

As for the APV estimation, available inertial sensors-based algorithms can be grouped in three main categories: human-gait model, direct integration, and abstraction model [11]. Human-gait model algorithms estimate walking speed using predefined human gait models that estimate the stride length essentially from the BCOM vertical displacement [12,13]. These algorithms require subject-specific model building and calibration (personalization); moreover, they tend to lack robustness, since stride length depends on features of the lower limb kinematics that cannot be captured by a human-gait model without additional measurements (e.g., the shank/thigh angle [12]). Direct integration algorithms perform the strap-down integration of the linear acceleration of the anatomical point of interest from null initial conditions of velocity, using kinematics constraints of walking in their formulation (i.e., zero-velocity update) [14,15]. These algorithms do not require personalization, and are generally accurate; however, they are restricted in the IMU positioning to feet or shanks for accurate detection of the zero-velocity conditions; IMU positioning to the pelvis was only proposed in [16]. Abstraction model algorithms use training via machine learning algorithms [17,18,19,20]. Although off-line training can be time consuming and would require a dense set of examples, yet they are fast and suited for real-time implementation [19,20]. These algorithms may suffer from limited generalization capabilities, although, in contrast with human-gait model algorithms, personalization is not mandatory; moreover, they are very flexible, in terms of sensor type and placement site [19].

Direct integration and abstraction model methods can perform equally well when compared with the same ground-truth data, at least in conditions of normal level walking and for an IMU positioning to feet [17]. The large variety of signals that can potentially be used as inputs to an abstraction model is the key asset of abstraction model methods, for the considerable freedom they give as for the choice of the IMU location. In principle, they can be applied to learn the kinematics of any point on the human body, provided that suitable reference data are available for learning [19].

In this paper we develop an abstraction model algorithm, based on the Bayesian Linear Regression (BLR) with Automatic Relevance Determination (ARD) [21,22]; the approach is to learn the underlying relationship between feature vectors from the shank IMU signals and the pelvis APV, without using the kinematics equation of motion, that may lead to drifted velocity estimates. Bayesian approaches to regression allow to handle conveniently problems of data over-fitting and model selection; in particular, the ARD-BLR comes with a built-in mechanism of feature selection, which enables the removal of irrelevant feature variables from the input space. Computational efficiency of training is another advantage of the ARD-BLR in our proposed application, as compared with other Bayesian methods, e.g., the Gaussian Process Regression [22,23].

In our approach, the feature vectors include, among other variables, the Fourier series coefficients computed from the shank IMU signals at each gait cycle; the feature vectors are mapped to the APV of that cycle (target) without requiring any kinematics constraint of walking. The Fourier series coefficients from the shank IMU are computed using the same method used to process the pelvis acceleration. The Fourier-based approach is then used to efficiently implement two different tasks (time integration and regression) and is applied to data from individual gait cycles, thus requiring a gait segmentation phases algorithm. In this paper, a Hidden Markov Model (HMM) is used to perform gait phases segmentation from the measured angular velocity of the shank. The HMM is capable of detecting the underlying periodicity of gait data and performs data-driven windowing without loss of time resolution [17].

## 2. Materials and Methods

### 2.1. Datasets

In this paper, we revisited two datasets available from our previous research. The Treadmill Walking (TW) dataset collects IMU and OMCS data from healthy subjects that performed trials of treadmill walking at preset speeds [10] (Table 1). The Overground Walking (OW) dataset collects IMU and OMCS data from healthy subjects that walked along a “figure-of-eight” pathway at their preferred speed [24] (Table 2).

In both datasets, the experimental setup required two IMUs that were attached to the lumbar spine (L5 level) and to the shank (above the right malleolus). Clusters of four retro-reflective markers were mounted on a small plastic support rigidly attached to each IMU. In the case of the TW dataset, three additional retro-reflective markers were available in correspondence of the heel, the first metatarsal head and the fifth distal phalanx of the instrumented foot. Standard precautions were taken for aligning the IMU axes approximately along the anatomical directions and securing the IMUs in place firmly [10,24].

### 2.2. IMU and OMCS Data Pre-Processing

**Figure 1:**
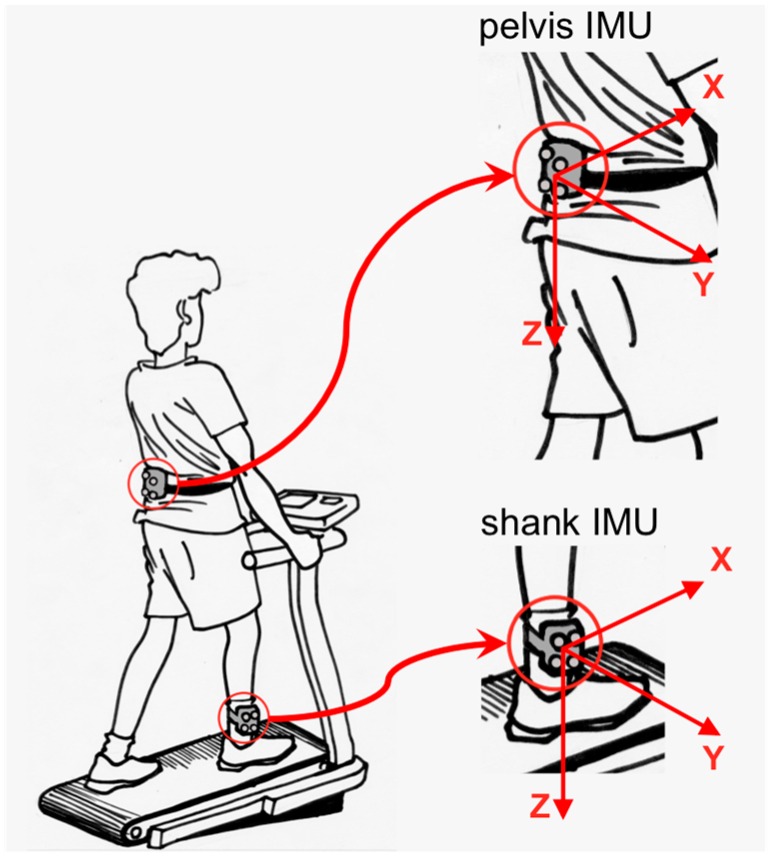
Unit Local Frames for the shank and pelvis Inertial Measurement Units (IMUs).

The procedures of IMU sensor calibration, frame registration, synchronization and conditioning of IMU and OMCS data streams were the same in the construction of both datasets [24]. The Cartesian coordinate systems fixed with the OMCS and an IMU were denoted, respectively, as the Global Earth-fixed Frame (with one axis aligned with Gravity) (GGF) and the Unit Local Frame (ULF). The orientation of the IMU axes were X: Antero-Posterior (AP) and positive forward; Y: Medio-Lateral (ML) and positive to the right; and Z: VerTical (VT) aligned with the direction of gravity and positive downwards, Figure 1.

Virtual markers were created in correspondence of the center of the tri-axial accelerometer chips, where the origins of the ULFs were located, by using the positions of the four retro-reflective markers associated to each IMU (Table 1 and Table 2).

### 2.3. Mathematical Processing

During steady-state level locomotion it is reasonable to assume that limb, or trunk displacement data can be modeled as quasi-periodic functions of time. Body segment displacement data have been obtained that can be accurately described through Fourier series that contain up to *M* = 6 significant harmonics [4,7]:

(1) $$f\left(t\right)=c_{0}+\overset{M}{\underset{i=1}{\sum }}\left(a_{i}\sin\left(i\frac{2\pi }{T}t\right)+b_{i}\cos\left(i\frac{2\pi }{T}t\right)\right)$$

where the DC component $c_{0}$ accounts for the effect of forward motion and the period T is the stride time. In this paper, the stride time is identified by the time elapsed between successive contacts of the same foot with the ground. The Fourier series can also be represented in the so-called phase-angle form:

(2) $$f\left(t\right)=c_{0}+\overset{M}{\underset{i=1}{\sum }}c_{i}\sin\left(i\frac{2\pi }{T}t+\varphi_{i}\right)$$

Suppose that f(t) describes the instantaneous linear velocity of a point on the human body in the progression direction. The DC component $c_{0}$ is identified with the APV of this point, with the cyclical component of velocity being described by the partial sum series in Equations (1) and (2).

**Figure 2:**
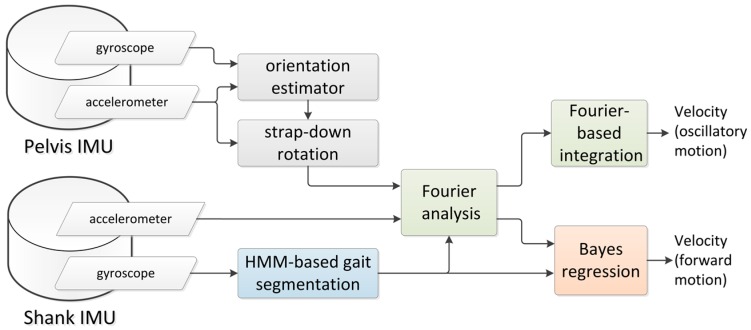
Computational flow of the proposed method.

Figure 2 reports the block diagram of the method proposed in this paper to estimate the instantaneous velocity of the pelvis. All computations were performed using functions written in MATLAB (The MathWorks, Natick, MA, USA).

### 2.4. Gait Phases Segmentation

The ML component of the angular velocity measured by the shank gyroscope was used to perform the gait phases segmentation. A four-state left-right Hidden Markov Model (HMM) was developed with two-dimensional emission vectors that included the measured angular velocity and its first time difference [25]. The emissions were modeled as Gaussian mixtures with three modes. The gait phases that were paired to model states were defined by consecutive occurrences of Foot Strike (FS), Flat Foot (FF), Heel Off (HO) and Toe Off (TO).

IMU and OMCS data from the TW dataset were used for HMM training. The data from the foot markers were used to generate the reference data for the FS, FF, HO and TO gait events in the *k*-th gait cycle, namely $t_{FS}\left(k\right),\;t_{FF}\left(k\right),\;t_{HO}\left(k\right),t_{TO}\left(k\right)$. Model parameters were trained in a supervised way, and testing was done using a Leave-One-Out-Subject (LOSO) validation study: one subject was tested with a model learnt using data from all other subjects; the training-testing procedure was then repeated, with all subjects being used once for testing.

### 2.5. Target Extraction

The nominal speed of the treadmill machine was the reference chosen for the APV (TW-APV_REF_) in the TW dataset. In the case of the OW dataset a different procedure was needed. A task-specific HMM was not trained, since, in contrast with the TW dataset, OMCS foot-marker data were not available (Table 2); rather, we used the HMM structure learnt using the TW dataset. The *X* and *Y* coordinates of the virtual marker at the pelvis (shank) were submitted to a first central difference method for numerical differentiation, after being low-pass filtered using a forward-backward second-order Butterworth filter (cut-off frequency: 3 Hz). The norm of the instantaneous velocity in the horizontal plane was then computed and averaged for each detected gait cycle, yielding the pelvis (shank) OW-APV_REF_.

### 2.6. Strap-Down Rotation

The Extended Kalman Filter (EKF) discussed in [24] was used to estimate the quaternion from the pelvis ULF to the GGF. The estimated quaternion was used to perform the strap-down rotation of the accelerometer output, so as to obtain the linear acceleration of the pelvis IMU. In the EKF design the magnetic sensor measurements were dismissed, although they were potentially helpful to stabilize the yaw estimate; this was done to meet the experimental setups that are typically used in studying the kinematics of pelvic motion [10].

### 2.7. Fourier Analysis for Estimating the Cyclical Component

Data from the pelvis IMU were submitted to Fourier analysis at each gait cycle (stride) [10]. In short, each linear acceleration component (namely: ML, VT, AP) of the *k*-th stride was analyzed and the Fourier series coefficients were computed up to *M* = 6:

(3) $$\begin{array}{l} acc_{\mathrm{ML}}^{k}=a_{ML0}\left(k\right)+\overset{M}{\underset{i=1}{\sum }}\left(a_{MLi}\left(k\right)\sin\left(i\frac{2\pi }{T_{k}}t\right)+b_{MLi}\left(k\right)\text{cos}\left(i\frac{2\pi }{T_{k}}t\right)\right)\\ acc_{\mathrm{VT}}^{k}=a_{VT0}\left(k\right)+\overset{M}{\underset{i=1}{\sum }}\left(a_{VTi}\left(k\right)\sin\left(i\frac{2\pi }{T_{k}}t\right)+b_{VTi}\left(k\right)\text{cos}\left(i\frac{2\pi }{T_{k}}t\right)\right)\\ acc_{\mathrm{AP}}^{k}=a_{AP0}\left(k\right)+\overset{M}{\underset{i=1}{\sum }}\left(a_{APi}\left(k\right)\sin\left(i\frac{2\pi }{T_{k}}t\right)+b_{APi}\left(k\right)\text{cos}\left(i\frac{2\pi }{T_{k}}t\right)\right) \end{array}$$

where $T_{k}$ is the duration of the *k*-th stride. The analytical integration of Equation (3) where $a_{\mathrm{ML}0}\left(k\right),\;a_{\mathrm{VT}0}\left(k\right)$ and $a_{\mathrm{AP}0}\left(k\right)\;$ were set to zero (equivalent to removing a constant term from the original stride linear acceleration data) led to the following expression of the mean-subtracted *k*-th stride velocity data:

(4) $$\begin{array}{l} vel_{\mathrm{ML}}^{k}=\frac{T_{k}}{2\pi }\overset{M}{\underset{i=1}{\sum }}\frac{1}{i}\left(-a_{MLi}\left(k\right)\cos\left(i\frac{2\pi }{T_{k}}t\right)+b_{MLi}\left(k\right)\sin\left(i\frac{2\pi }{T_{k}}t\right)\right)\\ vel_{\mathrm{VT}}^{k}=\frac{T_{k}}{2\pi }\overset{M}{\underset{i=1}{\sum }}\frac{1}{i}\left(-a_{VTi}\left(k\right)\cos\left(i\frac{2\pi }{T_{k}}t\right)+b_{VTi}\left(k\right)\sin\left(i\frac{2\pi }{T_{k}}t\right)\right)\\ vel_{AP}^{k}=\frac{T_{k}}{2\pi }\overset{M}{\underset{i=1}{\sum }}\frac{1}{i}\left(-a_{APi}\left(k\right)\cos\left(i\frac{2\pi }{T_{k}}t\right)+b_{APi}\left(k\right)\sin\left(i\frac{2\pi }{T_{k}}t\right)\right) \end{array}$$

It is noted that the mean subtraction in Equation (3) is implemented in recognition of practical difficulties in estimating the pelvis APV by time integration of noisy acceleration data [8].

One advantage of the Fourier regression (Equations (1) and (2)) is that, differently from, e.g., a polynomial regression, the coefficients computed up to a given order do not change when one is interested in adding further terms to the model (e.g., for achieving a better model fit to the original time function). Moreover, when dealing with raw data, effective smoothing can be achieved by using Fourier basis functions [26]. The Fourier analysis was performed using the methods of functional data analysis developed in [26], for the implementation of which a MATLAB toolbox is available.

### 2.8. Fourier Analysis for Estimating the APV

For the purpose of APV prediction, we propose to use the coefficients ${\left\{c_{i}\left(k\right)\right\}}_{i=1}^{M}$ of the Fourier series in the phase-angle form, which were computed using the *k*-th stride signals from the shank IMU. Six measurement channels were available (three for the acceleration, three for the angular velocity). The feature vector built at the *k*-th stride included, in addition to the Fourier harmonic coefficients for each measurement channel, the *k*-th stride time $T_{\mathrm{FS}}\left(k\right),$ and some additional temporal parameters of gait from the HMM:

(5) $$\left\{ \begin{array}{c} T_{\text{FS}}\left(k\right)=t_{FS}\left(k+1\right)-t_{FS}\left(k\right)\\ T_{\text{FF}}\left(k\right)=t_{FF}\left(k\right)-t_{FS}\left(k\right)\\ T_{\text{HO}}\left(k\right)=t_{HO}\left(k\right)-t_{FS}\left(k\right)\\ T_{\text{TO}}\left(k\right)=t_{TO}\left(k\right)-t_{FS}\left(k\right)\; \end{array}\right.$$

The feature vector had thus size *d* = 6*M* + 4. By preliminary testing, a good trade-off between fitting accuracy and APV predictive power was achieved by retaining the first two harmonic components of shank angular velocity and acceleration (i.e., M=2 and d=16).

### 2.9. Bayesian Linear Regression

The model was created from a training set of *N* observations $D={\left\{x\left(i\right),\;v\left(i\right)\right\}}_{i=1}^{N};\;x\left(i\right)\in {\mathbb{R}}^{d}$ is the feature vector and v(i) is the APV target paired to the *i*-th gait cycle. Henceforth, the feature vectors are denoted collectively by the N×D data matrix X, whose *n*-th row is $x_{n}^{T}\left(n=1,\ldots ,N\right)$, and the corresponding target values are given by the column vector $v={\left[v\left(1\right)\ldots v\left(N\right)\right]}^{T}$.

For some parameter vector **w**, the targets v(i) are given by adding noise to a linear combination of the input variables:

(6) $$\begin{array}{c} v\left(i\right)=f\left(x\left(i\right),\;w\right)+\varepsilon \left(i\right)\\ f\left(x\left(i\right),\;w\right)=w_{0}+\overset{d}{\underset{j=1}{\sum }}w_{j}x_{ji}=w^{T}x\left(i\right) \end{array}$$

where the residual ε(i) is modeled as a zero-mean Gaussian random variable with precision (inverse variance) α. When a bias weight $w_{0}$ or offset is included in the parameter (weight) vector w, as in Equation (6), the input vector is augmented with an additional element whose value is always one, which leads to the following expression of the N×(D+1) design matrix Φ:

(7) $$\Phi =\left[\begin{array}{cc} 1 & x_{1}^{T}\\ \vdots & \vdots \\ 1 & x_{N}^{T} \end{array}\right]$$

In the BLR approach a zero-mean Gaussian prior with precision $\beta_{i}$ is assigned to each parameter $w_{i}$:

(8) $$p\left(w_{i}\right)=N\left(w_{i}|0,\beta_{i}^{-1}\right)$$

Under the assumption of independence of the marginal distributions, the weight prior is written as follows:

(9) $$p\left(w|B\right)={\left(\frac{1}{2\pi }\right)}^{\left(d+1\right)/2}\overset{d}{\underset{i=0}{\prod }}\beta_{i}^{2}\exp\left(-\frac{\beta_{i}w_{i}^{2}}{2}\right)$$

where $B=\mathrm{diag}\left(\beta_{0},\ldots ,\beta_{d}\right)$.

The likelihood function of the target data is given by:

(10) $$p\left(v|X,w,\alpha \right)={\left(\frac{\alpha }{2\pi }\right)}^{N/2}\;\exp\left(-\frac{\alpha }{2}\|v-\Phi w{\|}^{2}\right)$$

The posterior distribution p(w|X, B , α) for the weights is Gaussian, with mean and covariance given by:

(11) $$\{\begin{array}{c} \mu =\alpha \;\Sigma \;\Phi^{T}v\\ \Sigma ={\left(B+\alpha \;\Phi^{T}\Phi \right)}^{-1} \end{array}$$

The values of the hyperparameters α, B are obtained by maximization of the log-marginal likelihood:

(12) $$\ln p\left(v|X,B,\alpha \right)=-\frac{1}{2}\left\{N\ln\left(2\pi \right)+\ln\left|C\right|+v^{T}C^{-1}v\right\}$$

where the N×N matrix C is written in the following form:

(13) $$C=\alpha^{-1}I+\Phi B^{-1}\Phi^{T}$$

The point estimates of B, α can be substituted back into Equation (11) to give an updated posterior distribution for the weights, from which re-compute the log-marginal likelihood. B and α can be thus iteratively updated for maximization [20]:

(14) $$\{\begin{array}{c} \beta_{i}=\frac{\gamma_{i}}{\mu_{i}^{2}}\\ \frac{1}{\alpha }=\frac{\|v-\Phi \mu {\|}^{2}}{N-\sum_{i}\gamma_{i}} \end{array}$$

where $\mu_{i}$ is the *i*-th component of the posterior mean μ defined by Equation (11) and the quantity $\gamma_{i}$ is defined by

(15) $$\gamma_{i}=1-\beta_{i}\Sigma_{ii}$$

in which $\Sigma_{ii}$ is the *i*-th diagonal component of the posterior covariance matrix Σ defined by Equation (11). The iterative process stops when a criterion of convergence is met, i.e., the largest change in the values of the hyperparameters is below a tolerance.

During re-estimation, some $\beta_{i}$’s can become very large, shrinking the corresponding posteriors $p\left(w_{i}|X,B,\alpha \right)$ to zero; this implies that the corresponding *i*-th column in Φ can be “pruned” (Automatic Relevance Determination (ARD)). In our implementation, the $\beta_{i}$’s that were 200 times larger than the data precision α were discarded, removing the corresponding feature variables from the input space [21].

The LOSO validation was conducted to evaluate the performance of each regression model. A difference exists in the way TW and OW datasets were managed in this regard: the LOSO validation of the HMM-based gait event detector and the LOSO validation of the ARD-BLR model were intertwined in the former case. Conversely, in the latter case the LOSO validation of the HMM was not performed, since the HMM structure emerging from analyzing the TW dataset was used.

### 2.10. Performance Assessment

Steady-state gait strides were considered as for the TW dataset; data from the first 30-s periods of recording were thus discarded; all gait strides were retained for analysis in the case of the OW dataset. The performance assessment for the cyclical component of velocity was based on the procedure devised for method comparison studies [27,28]. The Mean Difference (MD) and upper and lower Limit of Agreement (LA), namely MD ± 1.96 Standard Deviation (SD) of differences, were computed for the cyclical component of velocity in the ML, VT and AP directions, as it was obtained from IMU and OMCS data. Henceforth, the LA width is the difference between the upper and lower LAs. Scatter plots were produced to visualize differences between IMU and OMCS data against their mean.

The APV_BLR_ values during steady-state gait cycles were compared with the treadmill speed (TW-APV_REF_), yielding the estimation error $e_{ln}$ per speed condition. The index l run over the number of strides $L_{n}$ that were walked by the *n*-th participant in each walking trial. In the case of overground locomotion, the APV_BLR_ values were compared with the corresponding target values (pelvis or shank OW-APV_REF_).

The mean bias error (MBE) was the mean value of $e_{ln}$ where, before taking the mean over participants, the mean error per participant $m_{n}$ was computed [15]:

(16) $$\begin{array}{c} m_{n}=\frac{1}{L_{n}}\overset{L_{n}}{\underset{l=1}{\sum }}e_{ln}\\ \mathrm{MBE}=\frac{1}{N}\overset{N}{\underset{n=1}{\sum }}m_{n} \end{array}$$

The Root Mean Square Error (RMSE) was the root mean square value of $m_{n}$:

(17) $$\mathrm{RMSE}=\sqrt{\frac{1}{N}\overset{N}{\underset{n=1}{\sum }}m_{n}^{2}}$$

The Average Root Mean Square Error (ARMSE) was the average of the root mean square values $e_{n}$ of the estimation error $e_{ln}$, computed for each participant:

(18) $$\begin{array}{c} e_{n}=\sqrt{\frac{1}{L_{n}}\overset{L_{n}}{\underset{l=1}{\sum }}e_{ln}^{2}}\\ \mathrm{ARMSE}=\frac{1}{N}\overset{N}{\underset{n=1}{\sum }}e_{n} \end{array}$$

## 3. Results

The HMM-based gait event detector estimated the time occurrences of the FS, FF, HO and TO events. The mean, the SD and the Mean Absolute Value (MAV) of their difference from the OMCS reference values, averaged across subjects, are reported in Table 3. A total number of 3377 strides were analyzed; missed and additionally detected gait strides (deletions and insertions, respectively) were not observed. As for the OW dataset, mean = 7.0 ms, SD = 29.8 ms, and MAV = 37.1 ms were obtained by analyzing the difference between the FS time occurrences delivered by the HMM and the Zijlstra’s method [29], which was applied to the pelvis accelerometer. A total number of 753 strides were analyzed, without observing any erroneous event.

**Figure 3:**
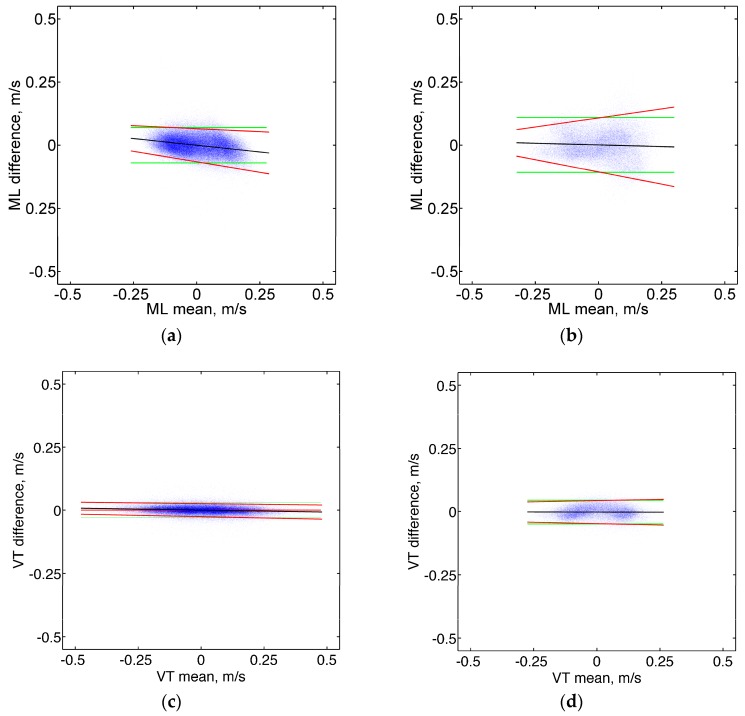

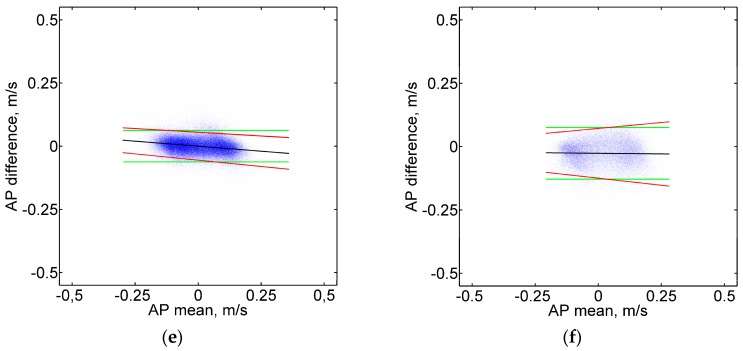
Bland Altman plots showing the difference between OMCS and proposed method as a function of their mean, for each component of velocity: ML (**a**,**b**); VT (**c**,**d**); and AP: (**e**,**f**). The plots in the panels (**a**,**c**,**e**) and (**b**,**d**,**f**) are produced from the TW and OW datasets, respectively. The line fitted to the plotted data is reported in black. The upper and lower limits of agreement based on the regression approach are reported in red. The green lines represent the constant limits of agreement computed without the regression-based correction.

Scatter plots of the difference between IMU and OMCS estimates of the cyclical component of the pelvis instantaneous velocity over their mean are reported in Figure 3 (data from all subjects and speed conditions were collapsed in producing the scatter plots).

Slight tendencies are observed for differences being increasingly negative with increasing mean value of the velocity; hence, compared to OMCS, the Fourier-based integration method slightly underestimated and overestimated for respectively negative and positive values of the velocity resolved in the GGF. Moreover, slight tendencies are observed for the spread of the differences to vary over the measurement range, which may produce non-constant LAs. In the attempt to refine the LA computation, the regression approach for non-uniform differences was applied [27]; the correction equations needed to compute the LA widths are reported in Table 4.

For each direction, a representative value of the LA width was finally obtained by taking the average of the widths that were computed over a range of velocity covering 95% of the measured values; the following values were obtained: ±0.07 m/s (ML component), ±0.03 m/s (VT component), and ±0.06 m/s (AP component) for the TW dataset; and ±0.10 m/s (ML component), ±0.05 m/s (VT component), and ±0.10 m/s (AP component) for the OW dataset. As a matter of comparison, the estimated vertical velocity profile of a trunk-mounted IMU during walking—reconstructed using strap-down integration [30]—was affected by root mean square errors of 0.08 m/s (on average), which translates, roughly, to LA widths of ±0.16 m/s.

**Figure 4:**
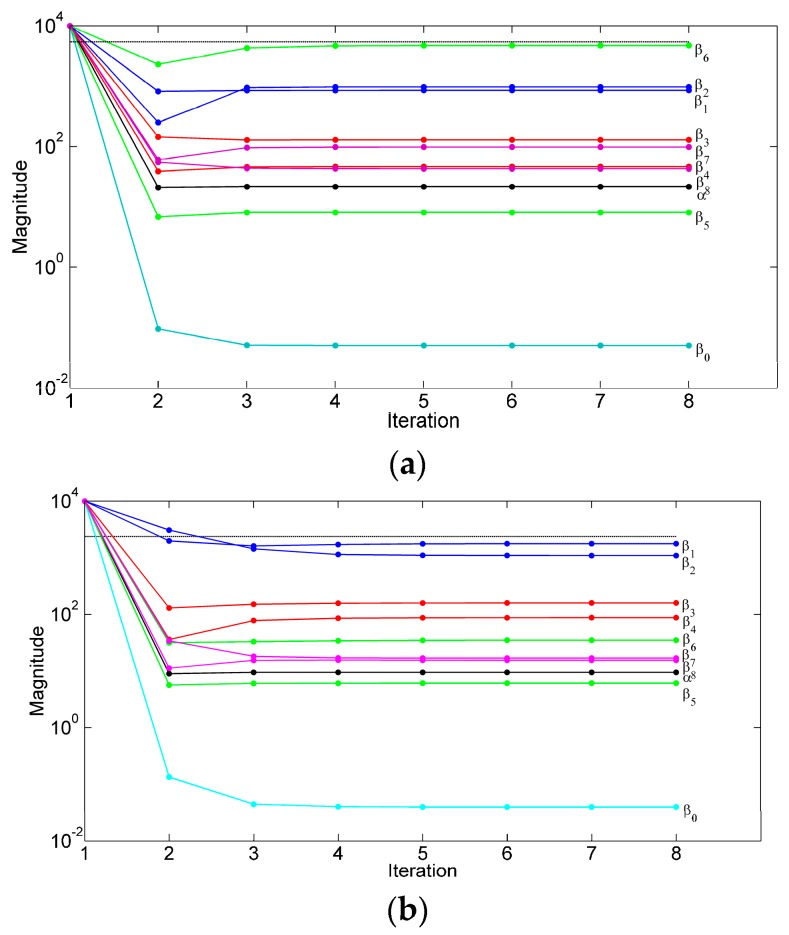
Time plots of the hyperparameters: (**a**) TW dataset; and (**b**) OW dataset. The priors of the stride time and stance duration were given precisions $\beta_{1}$ and $\beta_{2}$ , respectively; the priors of the ML, AP and VT components of the shank acceleration were given precisions $\beta_{3}$ – $\beta_{4}$, $\beta_{5}$ – $\beta_{6}$ and $\beta_{7}$ – $\beta_{8}$ , respectively; the Gaussian noise in the likelihood function was given precision α. The threshold for pruning the *i*-th column of the design matrix is depicted using the horizontal line in black.

The ARD process pruned some temporal features (i.e., $T_{\mathrm{FF}},\;T_{\mathrm{HO}}$) and all Fourier coefficients of the shank angular velocity; the stride time $T_{\mathrm{FS}}$ and the duration of the stance phase $T_{\mathrm{TO}}$ were retained, together with the Fourier series coefficients of the shank acceleration, yielding an input space of dimension *d* = 8, either in TW or OW conditions. The evolution of the hyperparameters in two representative runs of the ARD process is shown in Figure 4.

The performance metrics of the ARD-BLR are reported in Table 5 and Table 6, for TW and OW datasets, respectively (cross-validation was task-specific, namely training and testing were done using data from the same walking condition). As for the OW dataset, two different targets were considered, namely the pelvis and the shank OW-APV_REF_. Especially when walking along a curved path, the velocity fields changed across the body: Table 6 also reports the statistics of the pelvis and shank OW-APV_REF_ (minimum and maximum value, designated min and max, respectively, and the 25%, 50%, and 75% percentile, designated Q_1_, Q_2_ and Q_3_, respectively).

The final step of analysis consisted of training with one dataset and testing with the other dataset. The performance metrics of the ARD-BLR are reported in Table 7 and Table 8. Data in Table 7 were obtained when the regression model was trained with the OW dataset (target: shank OW-APV_REF_) and were tested with the TW dataset. Data in Table 8 concern the case in which the regression model was trained with the TW dataset and were tested with the OW dataset. The generalization capabilities of the method across the two walking conditions improved when using the pelvis OW-APV_REF_ as target for testing.

**Figure 5:**
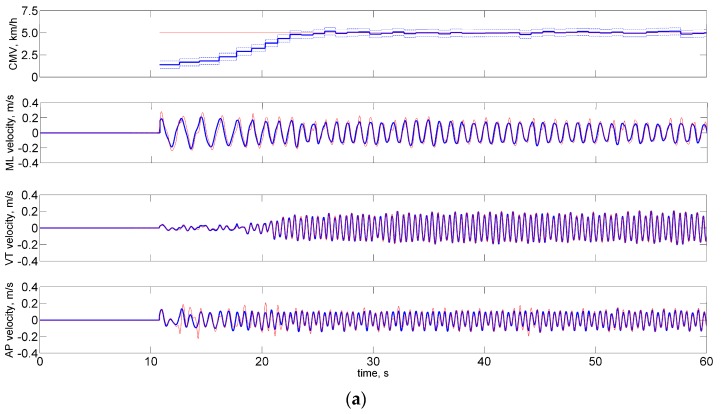

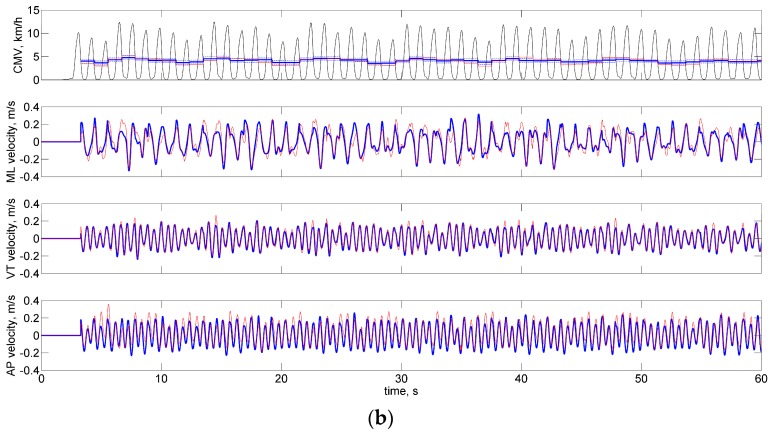
Representative time plots of the IMU and reference components of the pelvis instantaneous velocity, including transient and steady-state data: (**a**) TW dataset; and (**b**) OW dataset. The reference time functions are plotted in red; the time functions estimated with the proposed method are plotted in blue. In panel (**b**), the norm of the instantaneous velocity of the shank is plotted in black, and the bounds given by the predictive distribution are plotted using a dashed line.

Figure 5 shows representative examples of pelvis instantaneous velocity, when cross-validation was task-specific; transient stride data were also inspected for the treadmill-related example.

## 4. Discussion

The HMM-based gait phases segmentation method delivered accurate estimates of the gait events’ time occurrences, especially as for the FS and TO. FF and HO were detected with greater difficulty [31] (Table 3). The algorithm was capable of generalizing well across different tested subjects and walking conditions, although overground walking is known to be different from treadmill walking [32,33]; moreover, the participants recruited for constructing the two datasets were different people, and even the experimental setups were different in the two cases (Table 1 and Table 2). The Zijlstra’s method, which is widely used to detect gait strides [34], matched closely the HMM-based method as for the estimates of the FS events in conditions of overground walking.

As for the estimation of the cyclic component of velocity, the regression approach for non-uniform differences allowed accounting for slight systematic differences between the IMU and OMCS data, and to establish average LA widths across the measurement range (Table 4). It is noted that the agreement appears to be slightly looser in OW conditions, as compared with TW conditions, particularly for the horizontal components (i.e., ML and AP), compared with the vertical component (VT). This fact can be explained by the much larger measurement volume of the OMCS and by the longer duration of the OW trials. Both these factors make the procedure of aligning the ULF to the GGF in the direction of travel less accurate [24].

The ARD automatically removed feature variables that were irrelevant to the regression task. The same input space was considered to predict the APV in either TW or OW conditions, with one important difference. As shown in Figure 3, the ARD gave less importance to the temporal feature variables in OW conditions as compared with TW conditions (i.e., the corresponding priors were given higher precisions $\beta_{1}$ and $\beta_{2}$). Locomotion along a curved path is in fact a task that implies continuous deviation from straight-ahead locomotion, thereby requiring continuous adjustment of body movement [33]. For example, stride length is unchanged for the outer but decreases for the inner leg; however, the cadence is the same for both legs, in spite of the different length of the inner and outer strides. In other words, whilst the increase of the treadmill speed determined a marked reduction of stride time and duration of the stance phase [35], speed changes occurring during curved path locomotion were not accompanied by significant changes of either of them.

The predictive power of the input space slightly decreased in OW conditions compared with TW conditions when the validation was task-specific, Table 5 and Table 6. ARMSEs of less than 7.5%–8% were reported at the speed of 3 km/h, which fell at slightly less than 4% at velocities greater than 4 km/h. These results compare favorably with the state-of-the-art reported in the literature (e.g., ARMSE = 4%, overground walking with velocities 4–6 km/h, using a 16-dimensional input space of time- and frequency-domain feature variables from a foot IMU, in combination with a Linear Least Squares model [15]). Since, in contrast with RMSE, the ARMSE accounts for the intra-subject variability [15], it is not surprising that the ARMSE was larger than the RMSE in OW conditions, compared with TW conditions, due to the high walking regularity enforced by the treadmill machine.

When the regression model trained in OW conditions was applied to TW data for testing purposes (Table 7), the predictive performances degraded slightly compared with those obtained by the task-specific validation of Table 5. Using the regression model trained in TW conditions directly on OW data performed poorly, especially when the target for testing was the pelvis OW-APV_REF_, Table 8. Using the TW dataset, the shank acceleration signal features were used to predict the treadmill velocity, and the shank APV was matched to that velocity, Table 5. Since the forward component of the pelvis IMU motion was small (ideally, null) when walking on the treadmill machine, no effort was spent to learn the relationship between the input space and the pelvis APV. The use of the shank OW-APV_REF_ as target improved significantly the generalization abilities of the ARD-BLR across the two walking conditions. This was also true when the treadmill velocity was predicted using the regression model learnt from the OW data. As shown in Table 8, the best predictive performance were observed at 4 km/h, a value close to the mode of the distribution of the shank OW-APV_REF_ (Table 6); for the highest treadmill speeds, the ARD-BLR worked in extrapolation mode, which reflects in less predictive power. Under-estimation at 5 and 6 km/h treadmill speed were observed, which did not occur when validation was task-specific. ARMSEs of 9% were reported at the speed of 3 km/h, which fell at 5% at speeds greater than 4 km/h, implying a one percentage-point drop compared with when the validation was task-specific. Another factor, namely that the treadmill nominal speed might not perfectly match the actual walking speed, would also have contributed to this one percentage-point drop.

Finally, the good behavior of the proposed method is shown, qualitatively, in Figure 5; here the analysis started before the treadmill reached the preset velocity, although the ARD-BLR worked in extrapolation mode in these conditions. As for the Fourier-based method of integration, it is noted that the method could be successfully applied to data in the time interval between static upright posture and steady-state locomotion. The applicability of the proposed method during gait initiation is currently being studied in more detail, although we verified that the LA widths did not change appreciably when transient strides were accounted for in their calculation.

The model predictive power could be enhanced by subject-specific model calibration (i.e., biometric parameters, such as the height, are included in the input space) or personalization (i.e., few gait strides from a new subject for whom reference data are available are used to refine the model, thereby promoting a better match of estimates to the reference) [15]. Subject-specific calibration and personalization of the model were not considered in this paper and are left to our future work, which will be devoted to study mechanical energy changes of the BCOM in normal and pathologic walking using inertial motion sensors.

## 5. Conclusions

In this paper, an inertial sensor-based algorithm was developed with the aim of estimating the instantaneous velocity of an IMU attached to the pelvis during walking.

Under the assumption about the cyclical motion of human body parts during walking, the instantaneous velocity was modeled by the sum of two components, namely the cyclical component and the average progression velocity at each gait cycle. The algorithm made use of methods of Fourier harmonic analysis applied to pelvis and shank acceleration data to address two tasks: analytical time-integration of the linear acceleration, which enabled the estimation of the cyclical component, and regression using the ARD-BLR approach for estimating the average progression velocity. Analytical time-integration and regression required gait phases segmentation, which was efficiently done using an HMM-based gait event detector.

The inertial sensor-based algorithm was validated in conditions of treadmill and overground walking by healthy subjects. Analytical integration based on Fourier series coefficients was thus shown a useful approach to accurately estimate instantaneous velocity data from noisy acceleration measurements, whilst good generalizability of the ARD-BLR across different factors (namely, subjects, walking conditions, and IMU hardware) was demonstrated.

## Figures and Tables

**Table 1:** Setup and experimental protocol for the TW dataset [10].

**IMU**	WIMU (proprietary wireless battery-operated device) gyroscope (InvenSense ITG-3200, full range: ±2000°/s) accelerometer(Bosch BMA180, full range: ±4 g) magnetic sensor * (Honeywell HMC5843) barometric altimeter * (Bosch BMP085)
**OMCS**	five-camera system (Bonita B10, Oxford, UK)
**sampling frequencies**	IMU: 100 Hz OMCS: 100 Hz
**subjects**	*P* = 12 (6 male and 6 female), with age: 29.8 ± 7.8 years
**task**	2-min walking trials for each participant five trials, each at different speeds, from 3 to 7 km/h at steps of 1 km/h familiarization with treadmill walking allowed rest period of 5 s with the participants standing still in their upright posture before the start of each trial

* Although available, data from these sensors were not used in this paper.

**Table 2:** Setup and experimental protocol for the OW dataset [24].

**IMU**	OPAL (Opal, APDM Inc., Portland, OR, USA) gyroscope (full range: ±1500°/s) accelerometer (full range: ±6 g) magnetic sensor *
**OMCS**	nine-camera system (Vicon MX3, Oxford, UK)
**sampling frequencies**	IMU:128 Hz (digitally resampled at 100 Hz) OMCS: 100 Hz
**subjects**	*P* = 5 (3 male and 2 female), with age: 28.6 ± 5.1 years
**task**	level walking along a “figure of eight” pathway, (4 × 2 × 1.5) m walk at self-selected speed for 180 s rest period of 5 s with the participants standing still in their upright posture before the start of each trial

* Although available, data from these sensors were not used in this paper.

**Table 3:** Gait events statistics, in ms (difference between HMM-based and reference data).

	FS			FF			HO			TO		
Speed	Mean	SD	MAV	Mean	SD	MAV	Mean	SD	MAV	Mean	SD	MAV
3	14.2	22.3	20.7	134.0	059.9	134.2	−101.8	89.6	108.0	−18.8	22.9	24.8
4	14.7	18.1	19.4	−42.1	102.8	089.1	−57.5	72.1	068.3	−18.1	16.0	20.1
5	17.7	15.0	19.6	−17.2	086.5	054.6	−24.1	70.8	057.7	−12.0	14.5	14.8
6	12.8	18.3	17.8	−25.0	082.2	063.5	−16.1	75.8	068.9	–4.5	15.3	12.2
7	11.0	23.8	19.8	−36.8	071.1	064.0	−39.4	82.6	071.1	−7.7	27.7	15.5

**Table 4:** Regression equations of the difference (*D*) between the methods on the average (*A*) of the two methods, which provide the upper and lower LAs for each cyclical component of velocity.

**TW Dataset**	**Upper and Lower Limits of Agreement**
ML direction	*D* = −0.1065*A* − 0.0000 ± 2.46 (0.0242*A* + 0.0266)
VT direction	*D* = −0.0158*A* + 0.0001 ± 2.46 (0.0017*A* + 0.0105)
AP direction	*D* = −0.0795*A* + 0.0001 ± 2.46 (0.0083*A* + 0.0225)
**OW Dataset**	
ML direction	*D* = −0.0258*A* + 0.0013 ± 2.46 (0.0683*A* + 0.0435)
VT direction	*D* = −0.0015*A* − 0.0023 ± 2.46 (0.0084*A* + 0.0185)
AP direction	*D* = −0.0093*A* − 0.0262 ± 2.46 (0.0416*A* + 0.0398)

**Table 5:** Statistics of APV estimation for different treadmill speeds, in km/h (validation performed using the TW dataset).

Speed	MBE	RMSE	ARMSE
3	−0.094	0.237	0.240
4	−0.041	0.142	0.167
5	−0.057	0.183	0.193
6	−0.095	0.221	0.222
7	−0.007	0.231	0.252

**Table 6:** Statistics relevant to the target OW-APV_REF_ and to its estimation, in km/h (validation performed using the OW dataset; shank and pelvis indicate the site used to compute the target).

	Min	Q_1_	Q_2_	Q_3_	Max	MBE	RMSE	ARMSE
shank	2.1	3.2	3.7	4.1	5.9	−0.020	0.156	0.332
pelvis	2.6	3.1	3.4	3.7	4.3	−0.035	0.172	0.247

**Table 7:** Statistics of APV estimation for different treadmill speeds, in km/h (training done using the OW dataset, target: shank APV_REF_).

Speed	MBE	RMSE	ARMSE
3	−0.247	0.315	0.277
4	−0.138	0.174	0.189
5	−0.273	0.313	0.312
6	−0.202	0.307	0.276
7	−0.025	0.326	0.322

**Table 8:** Statistics of APV estimation in conditions of overground locomotion, in km/h (training done using the TW dataset; shank and pelvis indicate the site used as target for testing).

	MBE	RMSE	ARMSE
shank	−0.590	0.666	0.723
pelvis	−0.313	0.425	0.528

## Abbreviation

AP	Antero-posterior
APV	Average progression velocity
ARD	Automatic relevance determination
ARMSE	Average root mean square error
BCOM	Body center of mass
BLR	Bayesian linear regression
EKF	Extended Kalman filter
FF	Flat foot
FS	Foot strike
GGF	Global earth-fixed frame
HMM	Hidden Markov model
HO	Heel off
IMU	Inertial measurement unit
LA	Limits of agreement
LOSO	Leave-one-subject-out
MAV	Mean absolute value
MBE	Mean bias error
MD	Mean deviation
ML	Medio-lateral
OMCS	Optical motion capture system
OW	Overground walking
RMSE	Root mean square error
SD	Standard deviation
TO	Toe off
TW	Treadmill walking
ULF	Unit local frame
VT	Vertical
